# Supplementary material for: Vitamin D, B9, and B12 Deficiencies as Key Drivers of Clinical Severity and Metabolic Comorbidities in Major Psychiatric Disorders
Source: Nutrients. 2025 Mar 27;17(7):1167. doi: 10.3390/nu17071167 (PMC11990871; doi:10.3390/nu17071167)
Supplement: Supplementary file 1 [file nutrients-17-01167-s001.zip › nutrients-3524546-supplementary.pdf]

nutrients

MDPI

Supplementary Materials

Table S1. Hypovitaminosis B12 in schizophrenia

Schizophrenia	All	Univariate Analysis: Hypovitaminosis B12 No	Univariate Analysis: Hypovitaminosis B12 Yes	(p)	Multivariate Analysis: OR[a] (95% CI[b]) or standardized Betas	Multivariate Analysis: (p) adjusted
	N=295	N=292 (99.0%)	N=3 (1.0%)			
Sociodemographic						
Sex	82 (27.8%)	81 (27.7%)	1 (33.3%)			
Women						
Men	213 (72.2%)	211 (72.3%)	2 (66.7%)	0.625		
Age	34.30 (11.61)	32.07 (10.68)	44.58 (2.24)	0.003	0.116 (0.258-24.423)	0.045
65 years old and older	2 (0.7%)	2 (0.7%)	0 (0%)	0.980		
Working force status	34 (11.6%)	34 (11.7%)	0 (0%)	0.691		
Single	260 (88.1%)	258 (88.4%)	2 (66.7%)	0.316		
Education level	129 (61.4%)	128 (61.8%)	1 (33.3%)	0.331		
Psychiatric comorbidities						
ADHD[c]	4 (1.4%)	4 (1.4%)	0 (0%)	0.959		
Agoraphobia	40 (13.7%)	38 (13.1%)	2 (66.7%)	0.050	12.893 (1.106-150.297)	0.041
Generalized Anxiety Disorder	57 (19.5%)	57 (19.7%)	0 (0%)	0.521		
Panic Disorder	42 (14.3%)	42 (14.5%)	0 (0%)	0.628		
Social Phobia	43 (14.7%)	43 (14.8%)	0 (0%)	0.620		
PTSD[d]	7 (2.4%)	7 (2.4%)	0 (0%)	0.930		
Addictive comorbidities						
Tobacco smoking	165 (56.1 %)	164 (56.4%)	1 (33.3%)	0.408		
Tobacco (pack per year)	11.07 (14.20)	9.60 (12.21)	30.00 (-)	0.098	0.072 (-10.439-33.748)	0.299
Cannabis consumption	60 (20.3%)	60 (20.5%)	0 (0%)	0.504		
Alcohol Use Disorder	29 (10.0%)	29 (10.1%)	0 (0%)	0.729		
Clinical characteristics						
CDSS[e] score	4.27 (4.79)	4.39 (5.17)	3.67 (4.62)	0.810		
Depression (CDSS[e] cut-off)	67 (30.5%)	66 (30.4%)	1 (33.3%)	0.666		
SQoL-18[f] Index	54.10 (18.33)	52.65 (18.42)	51.27 (38.59)	0.956		
Fagerström score	5.08 (2.49)	4.93 (2.41)	6.00 (-)	0.657		
GAF[g] score	51.47 (15.70)	52.27 (15.85)	45.33 (10.50)	0.451		
Functionally Remitted (GAF[g])	69 (26.7%)	69 (27.1%)	0 (0%)	0.391		
STAI-YA[h] score	46.00 (14.49)	48.38 (11.75)	-	-		
MARS[i] score	6.45 (2.27)	6.24 (2.32)	7.00 (1.00)	0.572		
SF-36[j] physical health score	48.37 (9.59)	49.60 (8.07)	-	-		
SF-36[j] mental health score	32.54 (11.92)	32.93 (12.39)	-	-		
SBQ-R[k] score	7.67 (4.64)	7.84 (4.72)	12.00 (2.83)	0.216		
SBQ-R[k] cut-off	86 (47.5%)	84 (46.9%)	2 (100%)	0.224		
CGI[l] score	4.23 (1.15)	4.24 (1.15)	4.00 (1.00)	0.721		
UKU I[m]	4.82 (3.87)	5.45 (4.06)	2.33 (1.53)	0.187	-0.084 (-7.070-2.083)	0.284
UKU II[m]	1.22 (1.50)	1.14 (1.50)	0.33 (0.58)	0.355		
UKU III[m]	2.46 (2.50)	2.94 (2.53)	2.33 (2.52)	0.684		
UKU IV[m]	3.26 (3.62)	4.17 (3.93)	1.67 (1.53)	0.273		
Treatments						
Chlorpromazine equivalent dose	790.84 (756.36)	749.28 (708.15)	550.00 (492.44)	0.627		
Atypical antipsychotics	252 (85.4%)	249 (85.3%)	3 (100%)	0.622		
Typical antipsychotics	46 (15.6%)	45 (15.4%)	1 (33.3%)	0.400		

**Schizophrenia**	**All**	**Univariate Analysis**			**Multivariate Analysis**	
		**Hypovitaminosis B12**				
		**No**	**Yes**		**OR[a] (95% CI[b]) or standardized Betas**	**(p) adjusted**
	**N=295**	**N=292 (99.0%)**	**N=3 (1.0%)**	**(p)**		
Antipsychotics (typical and atypical)	262 (88.8%)	259 (88.7%)	3 (100.0%)	0.700		
Antidepressants	83 (28.1%)	81 (27.7%)	2 (66.7%)	0.192	4.325 (0.368-50.837)	0.244
Benzodiazepines	88 (29.8%)	87 (29.8%)	1 (33.3%)	0.656		
Mood Stabilizers	37 (12.5%)	36 (12.3%)	1 (33.3%)	0.332		
Physical Health						
Body Mass Index	25.87 (5.22)	25.66 (5.35)	28.23 (6.69)	0.409		
Obesity	58 (19.7%)	56 (19.2%)	2 (66.7%)	0.100	5.865 (0.514-66.963)	0.154
Total cholesterol	5.40 (8.59)	4.95 (1.14)	4.70 (2.20)	0.701		
LDL[n] cholesterol	3.11 (1.03)	3.05 (1.02)	2.55 (2.82)	0.843		
HDL[o] cholesterol	1.40 (0.60)	1.29 (0.41)	1.09 (0.35)	0.501		
hsCRP[p]	2.35 (2.31)	2.06 (2.08)	3.00 (3.57)	0.443		
Elevated hsCRP[p]	159 (58.7%)	157 (58.6%)	2 (66.7%)	0.629		
TSH[q]	2.32 (1.31)	2.34 (1.34)	3.07 (1.19)	0.346		
Prolactin	618.89 (877.87)	635.16 (925.30)	331.00 (136.12)	0.570		
Vitamin D	52.57 (30.40)	59.30 (32.47)	41.50 (2.12)	0.440		
Vitamin B9	14.41 (6.87)	14.50 (7.01)	10.36 (4.87)	0.405		
High Blood Pressure, diagnosed	9 (3.1%)	9 (3.1%)	0 (0%)	0.910		
Diabetes	11 (3.8%)	10 (3.5%)	1 (33.3%)	0.109	7.611 (0.623-92.909)	0.112
High Blood Pressure, measured	105 (35.8%)	104 (35.9%)	1 (33.3%)	0.707		
Hyperglycemia	32 (10.8%)	32 (11.0%)	0 (0%)	0.708		
Hypertriglyceridemia	81 (27.7%)	80 (27.7%)	1 (33.3%)	0.624		
Low HDL[o] cholesterol	88 (30.4%)	87 (30.3%)	1 (50.0%)	0.517		
High abdominal perimeter	170 (58.4%)	167 (58.0%)	3 (1.8%)	0.198	-	-
Metabolic Syndrome	79 (26.9%)	78 (26.8%)	1 (33.3%)	0.610		

* [a] Odd ratios. [b] confidence interval. [c] Attention Deficit and Hyperactivity Disorder. [d] Post-Traumatic Stress Disorder. [e] Calgary Depression Scale for Schizophrenia. [f] Schizophrenia Quality of Life – 18 items. [g] Global Assessment of Functioning. [h] State-Trait Anxiety Inventory – YA form. [I] Medication Adherence Rating Scale. [j] 36-items Short Form Health Survey Questionnaire. [k] Suicide Behaviors Questionnaire – Revised. [l] Clinical Global Impression. [m] Udvalg for Kliniske Undersøgelser. [n] Low-Density Lipoprotein. [o] High-Density Lipoprotein. [p] High-sensitivity C-Reactive Protein. [q] Thyroid-Stimulating Hormone. Significant values are in blue.

Table S2. Hypovitaminosis B12 in major depressive disorder

Major Depressive Disorder	All	Univariate Analysis			Multivariate Analysis	
		Hypovitaminosis B12			OR[a] (95% CI[b]) or standardized Betas	(p) adjusted
		No	Yes	(p)		
	N=312	N=302 (96.8%)	N=10 (3.2%)			
Sociodemographic						
Sex						
Women	176 (56.4%)	171 (56.6%)	5 (50.0%)			
Men	136 (43.6%)	131 (43.4%)	5 (50.0%)	0.752		
Age	43.50 (15.06)	43.56 (15.60)	33.89 (8.83)	0.007	-0.107 (-19.093-0.302)	0.058
65 years old and older	28 (9.0%)	28 (9.3%)	0 (0%)	0.385		
Working force status	78 (25.4%)	73 (24.6%)	5 (50.0%)	0.130	2.854 (0.796-10.233)	0.107
Single	141 (45.6%)	133 (44.5%)	8 (80.0%)	0.028	2.385 (0.462-12.315)	0.299
Education level	170 (64.6%)	163 (63.9%)	7 (87.5%)	0.160	3.540 (0.426-29.419)	0.242
Psychiatric comorbidities						
ADHD[c]	44 (14.1%)	43 (14.2%)	1 (10.0%)	0.576		
Agoraphobia	58 (18.7%)	55 (18.3%)	3 (30.0%)	0.282		
Generalized Anxiety Disorder	156 (50.5%)	151 (50.5%)	5 (50.0%)	1.000		
Panic Disorder	83 (26.8%)	80 (26.7%)	3 (30.0%)	0.529		
Social Phobia	61 (19.7%)	58 (19.3%)	3 (30.0%)	0.311		
PTSD[d]	32 (10.3%)	30 (10.0%)	2 (20.0%)	0.276		
Addictive comorbidities						
Tobacco smoking	124 (42.3%)	120 (42.3)	4 (44.4%)	0.577		
Tobacco (pack per year)	18.59 (15.77)	17.23 (15.87)	8.00 (4.00)	0.318		
Cannabis consumption	53 (17.8%)	52 (18.0%)	1 (11.1%)	0.503		
Alcohol Use Disorder	40 (13.7%)	37 (13.0%)	3 (33.3%)	0.110	3.586 (0.829-15.520)	0.088
Clinical characteristics						
CDSS[e] score	9.61 (5.56)	9.34 (5.98)	7.33 (3.22)	0.566		
Depression (CDSS[e] cut-off)	57 (69.5%)	55 (69.6%)	2 (66.7%)	0.670		
SQoL-18[f] Index	42.52 (18.61)	43.21 (19.33)	42.74 (23.95)	0.950		
Fagerström score	4.36 (3.23)	4.20 (3.18)	3.00 (5.20)	0.523		
GAF[g] score	55.06 (15.33)	56.67 (15.14)	49.75 (19.16)	0.370		
Functionally Remitted (GAF[g])	58 (33.3%)	57 (33.5%)	1 (25.0%)	0.593		
STAI-YA[h] score	50.30 (13.04)	49.23 (12.57)	43.29 (17.10)	0.226		
MARS[i] score	5.81 (2.32)	5.84 (2.34)	4.86 (1.86)	0.274		
SF-36[j] physical health score	47.12 (13.37)	47.83 (15.67)	53.00 (15.12)	0.515		
SF-36[j] mental health score	27.88 (13.54)	29.51 (15.61)	34.10 (16.05)	0.561		
SBQ-R[k] score	9.73 (5.31)	9.43 (5.21)	14.25 (1.71)	0.005	0.157 (-0.102-10.263)	0.055
SBQ-R[k] cut-off	91 (60.3%)	87 (59.2%)	4 (100.0%)	0.128	-	-
CGI[l] score	4.21 (1.25)	4.15 (1.28)	4.25 (1.26)	0.878		
UKU I[m]	6.74 (6.04)	7.34 (7.04)	10.00 (7.07)	0.597		
UKU II[m]	1.17 (1.71)	1.17 (1.81)	1.00 (1.41)	0.895		
UKU III[m]	3.29 (3.06)	3.58 (3.44)	1.00 (1.41)	0.295		
UKU IV[m]	4.26 (4.71)	4.73 (4.61)	4.50 (4.95)	0.944		
Treatments						
Chlorpromazine equivalent dose	80.04 (228.83)	82.33 (236.53)	50.00 (105.41)	0.667		
Atypical antipsychotics	51 (16.3%)	49 (16.2%)	2 (20.0%)	0.507		
Typical antipsychotics	9 (2.9%)	9 (3.0%)	0 (0%)	0.743		
Antipsychotics (typical and atypical)	57 (18.3%)	55 (18.2%)	2 (20.0%)	0.573		
Antidepressants	202 (64.7%)	198 (65.6%)	4 (40.0%)	0.094	0.502 (0.135-1.863)	0.303
Benzodiazepines	91 (29.2%)	88 (29.1%)	3 (30.0%)	0.597		
Mood Stabilizers	8 (2.6%)	8 (2.6%)	0 (0%)	0.768		

**Major Depressive Disorder**	**All**	**Univariate Analysis**			**Multivariate Analysis**	
		**Hypovitaminosis B12**			**OR[a] (95% CI[b]) or standardized Betas**	**(p) adjusted**
		**No**	**Yes**	**(p)**		
	**N=312**	**N=302 (96.8%)**	**N=10 (3.2%)**			
Physical Health						
Body Mass Index	25.45 (5.99)	25.41 (6.11)	26.43 (5.65)	0.623		
Obesity	63 (20.3%)	60 (19.9%)	3 (33.3%)	0.269		
Total cholesterol	5.20 (1.16)	5.15 (1.11)	4.56 (1.03)	0.098	-0.054 (-0.996-0.323)	0.316
LDL[n] cholesterol	3.16 (1.06)	3.11 (1.03)	2.43 (0.83)	0.050	-0.077 (-1.059-0.174)	0.159
HDL[o] cholesterol	1.53 (0.47)	1.54 (0.47)	1.58 (0.30)	0.809		
hsCRP[p]	2.11 (2.26)	2.01 (2.31)	2.14 (1.91)	0.859		
Elevated hsCRP[p]	151 (51.2%)	145 (51.2%)	6 (60.0%)	0.414		
TSH[q]	2.15 (1.66)	1.99 (1.11)	2.37 (0.95)	0.281		
Prolactin	340.79 (365.05)	325.64 (293.08)	280.75 (115.87)	0.666		
Vitamin D	64.96 (32.49)	69.00 (31.68)	71.44 (40.74)	0.822		
Vitamin B9	16.22 (9.27)	16.40 (9.43)	13.03 (6.98)	0.264		
High Blood Pressure, diagnosed	36 (11.6%)	36 (12.0%)	0 (0%)	0.287		
Diabetes	14 (4.5%)	14 (4.7%)	0 (0%)	0.627		
High Blood Pressure, measured	120 (38.7%)	116 (38.7%)	4 (40.0%)	0.587		
Hyperglycemia	41 (13.2%)	40 (13.3%)	1 (10.0%)	0.611		
Hypertriglyceridemia	59 (19.2%)	57 (19.2%)	2 (20.0%)	0.604		
Low HDL[o] cholesterol	51 (16.7%)	51 (17.2%)	0 (0%)	0.157	-	-
High abdominal perimeter	183 (61.2%)	179 (61.7%)	4 (44.4%)	0.239		
Metabolic Syndrome	58 (19.0%)	57 (19.3%)	1 (10.0%)	0.404		

* [a] Odd ratios. [b] confidence interval. [c] Attention Deficit and Hyperactivity Disorder. [d] Post-Traumatic Stress Disorder. [e] Calgary Depression Scale for Schizophrenia. [f] Schizophrenia Quality of Life – 18 items. [g] Global Assessment of Functioning. [h] State-Trait Anxiety Inventory – YA form. [I] Medication Adherence Rating Scale. [j] 36-items Short Form Health Survey Questionnaire. [k] Suicide Behaviors Questionnaire – Revised. [l] Clinical Global Impression. [m] Udvalg for Kliniske Undersøgelser. [n] Low-Density Lipoprotein. [o] High-Density Lipoprotein. [p] High-sensitivity C-Reactive Protein. [q] Thyroid-Stimulating Hormone. Significant values are in blue.

Table S3. Hypovitaminosis B12 in bipolar disorder

Bipolar Disorder	All	Univariate Analysis – Hypovitaminosis B12			Multivariate Analysis	
		No	Yes	(p)	OR[a] (95% CI[b]) or standardized Betas	(p) adjusted
	N=83	N=81 (97.6%)	N=2 (2.4%)			
Sociodemographic						
Sex						
Women	50 (60.2%)	49 (60.5%)	1 (50.0%)			
Men	33 (39.8%)	32 (39.5%)	1 (50.0%)	0.640		
Age	45.37 (14.10)	45.03 (13.91)	40.57 (8.77)	0.654		
65 years old and older	5 (6.0%)	5 (6.2%)	0 (0%)	0.882		
Working force status	18 (21.7%)	17 (21.0%)	1 (50.0%)	0.389		
Single	38 (45.8%)	36(44.4%)	2(100%)	0.207		
Education level	50 (70.4%)	48 (69.6%)	2 (100%)	0.493		
Psychiatric comorbidities						
ADHD[c]	4 (4.8%)	4 (4.9%)	0 (0%)	0.905		
Agoraphobia	20 (24.7%)	18 (22.8%)	2 (100%)	0.059	-	-
Generalized Anxiety Disorder	38 (46.9%)	36 (45.6%)	2(100%)	0.217		
Panic Disorder	20 (24.7%)	20 (25.3%)	0 (0%)	0.565		
Social Phobia	25 (30.9%)	25 (31.6%)	0 (0%)	0.475		
PTSD[d]	5 (6.2%)	5 (6.3%)	0 (0%)	0.880		
Addictive comorbidities						
Tobacco smoking	37 (49.3%)	37 (50.7%)	0 (0%)	0.253		
Tobacco (pack per year)	20.07 (14.94)	15.10 (12.04)	-	-		
Cannabis consumption	16 (21.9%)	15 (21.1%)	1 (50.0%)	0.393		
Alcohol Use Disorder	14 (19.4%)	14 (20.0%)	0 (0%)	0.647		
Clinical characteristics						
CDSS[e] score	7.21 (5.95)	6.63 (5.92)	-	-		
Depression (CDSS[e] cut-off)	14 (46.7%)	14 (46.7%)	-	-		
SQoL-18[f] Index	43.62 (21.68)	43.97 (22.41)	46.99 (14.16)	0.851		
Fagerström score	4.81 (3.23)	4.63 (3.47)	-	-		
GAF[g] score	58.37 (14.43)	59.29 (14.21)	65.00 (28.28)	0.589		
Functionally Remitted (GAF[g])	27 (47.4%)	26 (47.3%)	1 (50.0%)	0.727		
STAI-YA[h] score	47.92 (16.09)	49.14 (13.34)	-	-		
MARS[i] score	6.10 (2.32)	6.07 (2.21)	7.00 (1.41)	0.558		
SF-36[j] physical health score	45.91 (13.61)	44.76 (14.49)	50.03 (8.45)	0.613		
SF-36[j] mental health score	29.73 (15.30)	28.75 (15.90)	33.58 (14.74)	0.673		
SBQ-R[k] score	10.01 (5.40)	10.00 (5.42)	8.00 (4.24)	0.609		
SBQ-R[k] cut-off	38 (67.9%)	37 (68.5%)	1 (50.0%)	0.544		
CGI[l] score	3.96 (1.19)	3.84 (1.25)	3.50 (0.71)	0.705		
UKU I[m]	6.21 (4.98)	6.33 (4.87)	-	-		
UKU II[m]	1.00 (1.27)	0.91 (0.95)	-	-		
UKU III[m]	3.30 (3.10)	3.33 (3.30)	-	-		
UKU IV[m]	4.95 (4.66)	6.12 (4.82)	-	-		
Treatments						
Chlorpromazine equivalent dose	175.16 (345.90)	211.11 (394.45)	225.00 (35.36)	0.961		
Atypical antipsychotics	29 (34.9%)	27 (33.3%)	2 (100%)	0.119	-	-
Typical antipsychotics	3 (3.6%)	3 (3.7%)	0 (0%)	0.929		
Antipsychotics (typical and atypical)	30 (36.1%)	28 (34.6%)	2 (100%)	0.128	-	-
Antidepressants	50 (60.2%)	48 (59.3%)	2 (100%)	0.360		
Benzodiazepines	26 (31.3%)	25 (30.9%)	1 (50.0%)	0.531		
Mood Stabilizers	32 (38.6%)	35 (39.5%)	0 (0%)	0.375		

**Bipolar Disorder**	**All**	**Univariate Analysis**			**Multivariate Analysis**	
		**Hypovitaminosis B12**			**OR[a] (95% CI[b]) or standardized Betas**	**(p) adjusted**
		**No**	**Yes**	**(p)**		
	**N=83**	**N=81 (97.6%)**	**N=2 (2.4%)**			
Physical Health						
Body Mass Index	25.52 (5.45)	25.62 (5.11)	31.57 (0.49)	0.106	0.188 (-0.945-13.288)	0.088
Obesity	17 (21.0%)	15 (19.0%)	2 (100%)	0.042	-	-
Total cholesterol	5.33 (1.10)	5.32 (1.09)	5.94 (2.37)	0.443		
**LDL[n] cholesterol**	**3.18 (1.02)**	**3.25 (1.07)**	**1.64 (-)**	**0.145**	**-0.258 (-3.509—0.221)**	**0.027**
HDL[o] cholesterol	1.53 (0.50)	1.52 (0.48)	1.07 (0.09)	0.183	-0.131 (-1.027-0.223)	0.204
hsCRP[p]	1.92 (1.95)	1.85 (1.89)	3.05 (0.35)	0.375		
Elevated hsCRP[p]	40 (52.6%)	38 (51.4%)	2 (100%)	0.274		
TSH	2.30 (1.41)	2.45 (1.48)	2.74 (0.95)	0.780		
Prolactin	269.61 (209.20)	270.79 (193.80)	260.00 (12.73)	0.938		
Vitamin D	59.21 (27.56)	61.55 (27.06)	24.00 (-)	0.172	-0.160 (-92.326-17.414)	0.178
Vitamin B9	17.28 (8.71)	17.41 (8.66)	8.66 (5.44)	0.160	-0.148 (-20.595-4.507)	0.186
High Blood Pressure, diagnosed	6 (7.3%)	6 (7.5%)	0 (0%)	0.858		
Diabetes	1 (1.2%)	1 (1.2%)	0 (0%)	0.976		
High Blood Pressure, measured	30 (36.1%)	29 (35.8%)	1 (50.0%)	0.595		
Hyperglycemia	6 (7.3%)	6 (7.5%)	0 (0%)	0.858		
Hypertriglyceridemia	22 (27.2%)	20 (25.3%)	2 (100%)	0.071	-	-
Low HDL[o] cholesterol	20 (24.7%)	19 (24.1%)	1 (50.0%)	0.435		
High abdominal perimeter	53 (66.2%)	51 (65.4%)	2 (100%)	0.436		
Metabolic Syndrome	14 (17.1%)	13 (16.2%)	1 (50.0%)	0.314		

* [a] Odd ratios. [b] confidence interval. [c] Attention Deficit and Hyperactivity Disorder. [d] Post-Traumatic Stress Disorder. [e] Calgary Depression Scale for Schizophrenia. [f] Schizophrenia Quality of Life – 18 items. [g] Global Assessment of Functioning. [h] State-Trait Anxiety Inventory – YA form. [I] Medication Adherence Rating Scale. [j] 36-items Short Form Health Survey Questionnaire. [k] Suicide Behaviors Questionnaire – Revised. [l] Clinical Global Impression. [m] Udvalg for Kliniske Undersøgelser. [n] Low-Density Lipoprotein. [o] High-Density Lipoprotein. [p] High-sensitivity C-Reactive Protein. [q] Thyroid-Stimulating Hormone. Significant values are in blue.
